# Brazilian Butt Lift Gone Wrong: A Case Series of Non-Tuberculous Mycobacterial Gluteal Infection After Brazilian Butt Lifts

**DOI:** 10.7759/cureus.49881

**Published:** 2023-12-03

**Authors:** Jared J Bies, Jesse C Allen, Zahra E Barsi, Mariam Hassan, Swathi Prakash, Mateo Porres-Aguilar, Armando Meza, Diego P Peralta

**Affiliations:** 1 Internal Medicine, Texas Tech University Health Sciences Center El Paso, El Paso, USA; 2 Infectious Diseases, Texas Tech University Health Sciences Center El Paso Paul L. Foster School of Medicine, El Paso, USA; 3 Division of Infectious Diseases, Texas Tech University Health Sciences Center El Paso, El Paso, USA

**Keywords:** erythromycin resistance methylase, amikacin, cefoxitin, tigecycline, multi-drug resistant bacteria, gluteal augmentation surgery, gluteal augmentation, mycobacterium abscessus complex, wound infections, nontuberculous mycobacteria (ntm)

## Abstract

Cosmetic surgeries are very popular and glamorized by the mainstream media and celebrities. Many individuals perceive certain bodily features as appealing for physical attraction and will attempt to obtain these features by surgery. However, these surgeries are not without risk, and significant consequences can occur if not performed by qualified medical professionals under sterile procedures. The authors present novel cases of two healthy young female patients who underwent a Brazilian butt lift (BBL) procedure a week apart by the same plastic surgeon in Mexico and developed dark painful lesions secondary to *Mycobacterium abscessus *(*M. abscessus*), a multidrug-resistant non-tuberculous mycobacterium (NTM). The literature review shows a paucity of data concerning NTM infections via surgical procedures of this type.

The first case was of a 31-year-old woman who underwent a BBL and presented with bilateral dark painful buttock lesions weeks later. The patient returned to the plastic surgeon, who drained some lesions and prescribed oral antibiotics. The patient’s clinical status continued to deteriorate and presented to the hospital for further assessment. The patient was initially started on broad-spectrum antibiotic therapy. The patient was found to have an HIV infection with a relatively preserved CD4 lymphocyte count and was started on antiretroviral therapy (ART). Intraoperative excisional tissue sample cultures grew *M. abscessus*. The patient was started on empiric tigecycline, cefoxitin, and linezolid. Preliminary culture susceptibilities showed resistance to linezolid. Linezolid was discontinued, amikacin was started, and cefoxitin and tigecycline were continued. Tigecycline, cefoxitin, and amikacin were continued and final susceptibilities showed sensitivity to the current treatment. The patient received a total of four months of treatment with tigecycline, cefoxitin, and amikacin. The second case was of a 28-year-old woman who underwent a BBL a week after the first patient by the same surgeon and developed multiple gluteal and body abscesses. The patient underwent bilateral thigh and gluteal, right chest wall, and breast surgical debridements with intraoperative cultures at a different hospital facility, which grew *M. abscessus*. Susceptibilities were not performed there. The patient was transferred to our facility for further care. Intraoperative cultures remained negative, and the patient was treated with a six-month course of tigecycline, cefoxitin, and amikacin.

## Introduction

A Brazilian butt lift (BBL), also known as gluteal fat augmentation, has been performed by Brazilian plastic surgeons for more than 30 years [1]. It is a procedure where fat is harvested by a closed-system liposuction technique, separated by gravity, and injected into the gluteal region using a peristaltic pump and reticulating basket cannulas [2]. The increasing popularity of this procedure, with its associated complications, has made experts associate it with the highest risk of mortality of any esthetic procedure [1]. The most common post-procedural complications are fat or pulmonary embolism (PE) and deep vein thrombosis (DVT), along with instances of skin and soft tissue infections [3]. A study including 853 board-certified plastic surgeon members of the Brazilian Society of Plastic Surgery demonstrated that certain techniques were correlated with fewer postoperative complications [1]. This study showed how the risk of complications from this procedure is minimal when performed by appropriately trained surgeons but can have disastrous outcomes if performed in settings with poor infection control practices. These surgeries can lead to complicated postoperative infections that require extensive surgical interventions and lengthy antibiotic course regimens if poor sterilization techniques are used.

## Case presentation

Case 1

A 31-year-old woman underwent a BBL procedure in Mexico. She subsequently developed fever, chills, and dark-colored lesions on her gluteal muscle area two weeks after the initial procedure. The patient returned to her plastic surgeon, who drained several lesions and prescribed levofloxacin and clindamycin without any noticeable improvement. The patient continued to deteriorate clinically and presented to the hospital for further assessment five weeks after the initial surgery.

**Figure 1:**
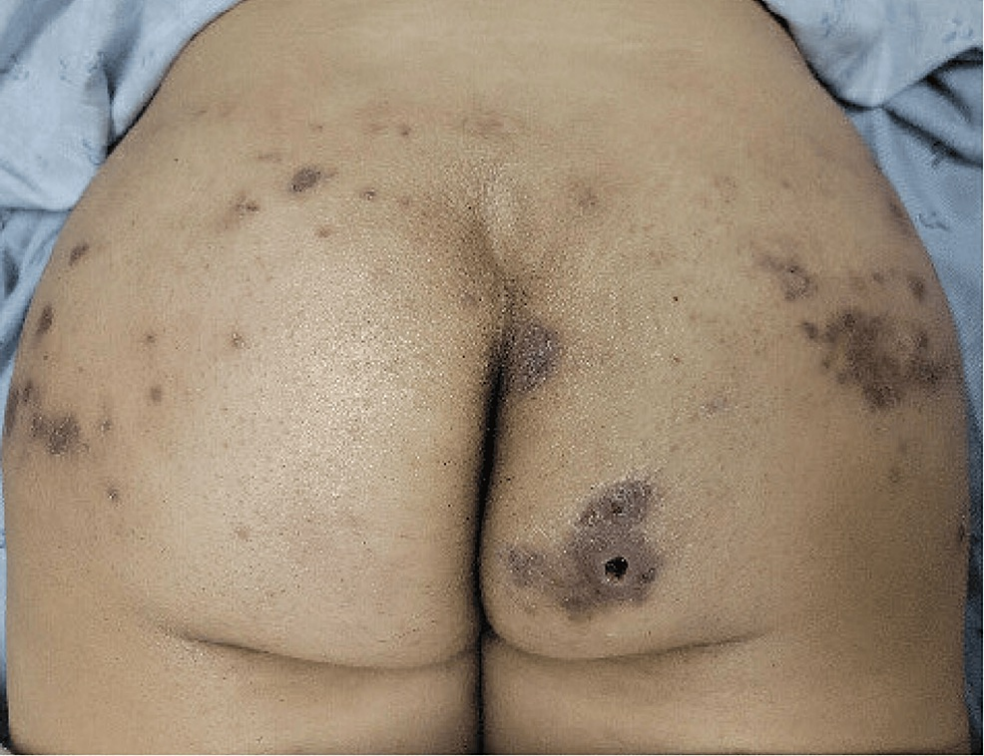
Initial bilateral gluteal lesions.

On presentation, the patient was afebrile (37.5°C), with tachycardia of greater than 140 beats per minute, dyspneic with oxygen saturation of 96% on room air, with painful lesions to bilateral gluteal areas, and bilateral lower-extremity swelling. Examination revealed several tender, dark-colored, and indurated lesions (Figure 1).

Initial laboratory work-up revealed leukocytosis, anemia, thrombocytosis, and a positive rapid HIV test (Table 1).

**Table 1:** Initial laboratory work-up results.

Tests	Results	Reference Range
White blood cell count (WBC)	11.49 x 10^3^/uL	4.50-11.03 x 10^3^/uL
Red blood cell count (RBC)	4.25 x 10^6^/uL	3.50-5.50 x 10^6^/uL
Hemoglobin	10.8 g/dL	12.0-15.0 g/dL
Platelets	490 x 10^3^/uL	150-450 x 10^3^/uL
Sodium, serum	137 mmol/L	135-145 mmol/L
Potassium, serum	4.4 mmol/L	3.5-5.1 mmol/L
Chloride, serum	101 mmol/L	98-107 mmol/L
Bicarbonate	26 mmol/L	22-30 mmol/L
Glucose	105 mg/dL	74-106 mg/dL
Blood urea nitrogen, serum	12 mg/dL	7-17 mg/dL
Magnesium, serum	1.7 mg/dL	1.6-2.3 mg/dL
Phosphorus, serum	5.2 mg/dL	2.5-4.5 mg/dL
Thyroid-stimulating hormone	7.240 MIU/L	0.465-4.680 MIU/L
Glycated hemoglobin A1c	5.0%	<5.7%
HIV ½ rapid 4th generation	Reactive	Nonreactive

**Figure 2:**
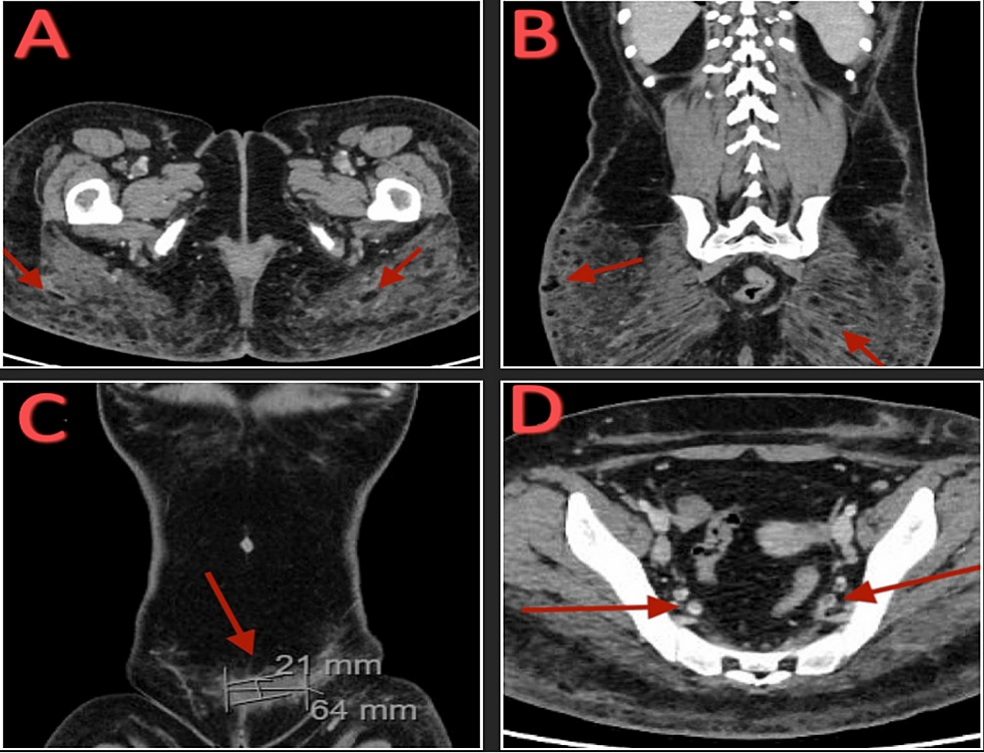
CT abdomen and pelvis with contrast. (A) Transverse view of small subcutaneous fluid collections and diffuse subcutaneous thickening and fat stranding in bilateral gluteal soft tissues. (B) Coronal view of small subcutaneous fluid collections and diffuse subcutaneous thickening and fat stranding in bilateral gluteal soft tissues. (C) Coronal view of a small subcutaneous fluid collection measuring 21 mm × 64 mm. (D) Filling defect in bilateral common femoral veins.

ECG showed sinus tachycardia, nonspecific T wave abnormality, and T wave inversions evident in anterior leads. Bedside point-of-care ultrasound (POCUS) was negative for both right ventricular (RV) strain and McConnell’s sign. CT abdomen and pelvis with contrast showed diffuse subcutaneous thickening, fat stranding in bilateral gluteal soft tissues, a small subcutaneous fluid collection measuring 21 mm × 64 mm, and bilateral filling defects in the common femoral veins (Figure 2).

**Figure 3:**
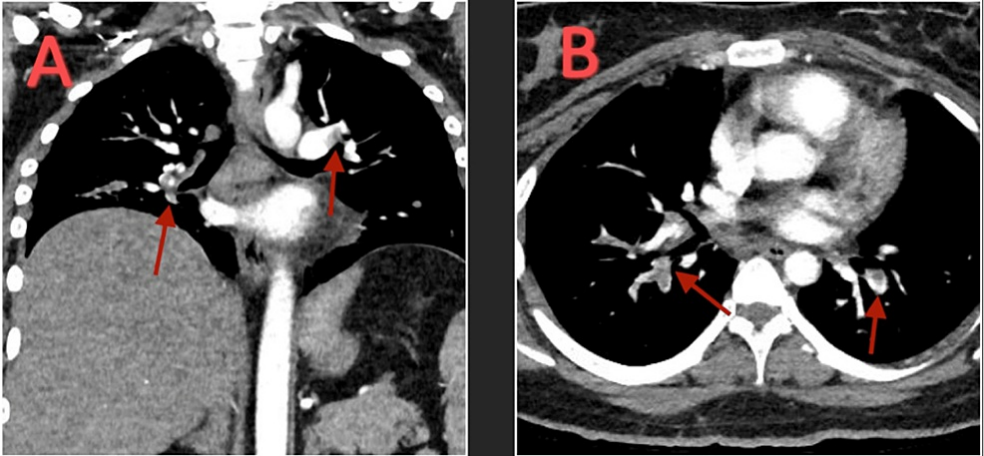
CTA chest with contrast. (A) Coronal view showing filling defects at the right interlobar artery and within the lobar and segmental branches of the right lower lobe. Left side filling defects with thrombus extending into the lobe and segmental branches of the lower lobe. (B) Transverse view showing similar filling defects. CTA: CT angiography.

Venous Doppler showed bilateral femoral DVTs. CT angiography (CTA) of the chest with contrast showed filling defects at the right interlobar artery, within the lobar and segmental branches of the right lower lobe, and left side filling defects with thrombus extending into the lobe and segmental branches of the lower lobe (Figure 3).

Formal transthoracic echocardiogram (TTE) showed normal left ventricular (LV) size, LV ejection fraction (EF) of 55-60%, RV normal size and function, and no signs of pericardial effusion.

**Figure 4:**
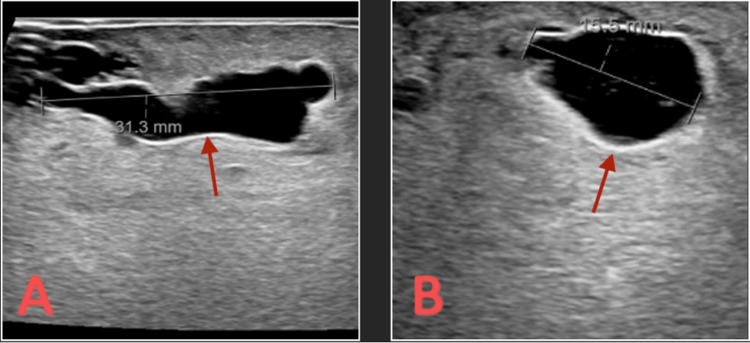
(A) Soft tissue ultrasound showing 31.1 mm fluid collection in the right glute. (B) Soft tissue ultrasound showing 15.5 mm fluid collection in the right glute.

The patient was initially started on empiric antibiotics with vancomycin and piperacillin-tazobactam. The patient was started on therapeutic low-molecular-weight heparin for extensive PEs and DVTs. A non-vascular ultrasound of bilateral lower extremities was significant for multiple fluid collections, with the largest ones measuring 31.1 mm and 15.5 mm in the right gluteus (Figure 4).

The patient underwent incision and drainage (I&D) and an excisional biopsy of the right and left gluteal areas, 4.2 x 3 cm and 4.5 x 2 cm, respectively. MRSA nares screen returned negative, and the patient’s antibiotic treatment was de-escalated to only piperacillin-tazobactam. Additional labs demonstrated an HIV PCR with 27,7000 copies/mL and a CD4+ count of 382 cells/mcL (Table 2).

**Table 2:** Additional HIV characterization labs.

Tests	Results	Reference Range
Lymphocytes	2246 cells/mcL	850-3,900 cells/mcL
CD3, Absolute	1621 cells/mcL	840-3,060 cells/mcL
CD3, Percentage	72%	57-85%
CD4, Absolute	382 cells/mcL	490-1,740 cells/mcL
CD4, Percentage	17%	30-61%
CD8, Absolute	1241 cells/mcL	180-1,170 cells/mcL
CD8, Percentage	55%	12-41%
CD4/CD8 ratio	0.31	0.86-5.00
HIV-1 RNA, PCR	277,000 copies/mL	0 copies/mL
HIV-1 antibody	Positive	Negative
HIV-2 Antibody	Negative	Negative

**Figure 5:**
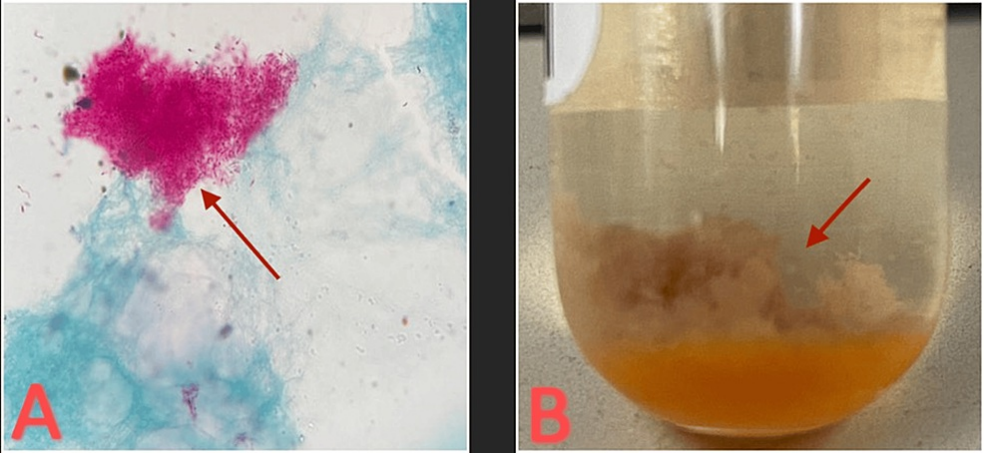
(A) Kinyoun stain positive for acid-fast bacilli. (B) Mycobacterium abscessus growth in liquid media.

These results indicated a pre-surgical infection and stage 2 HIV infection. The patient was started on antiretroviral therapy (ART) with dolutegravir and lamivudine. Intraoperative samples from initial I&D were positive for 1-9 acid-fast bacilli (AFB) on Kinyoun stain, and growth in liquid medium was observed within the first week, suggestive of a rapidly growing nontuberculous mycobacterium (NTM) (Figure 5).

Cultures were sent to Quest Diagnostics, the main hospital reference laboratory, for initial identification and susceptibilities. Another sample was also sent to National Jewish Health for comprehensive drug susceptibility testing. Repeat CT of the abdomen and pelvis with contrast showed new multiple soft tissue defects and ulcerations representing abscesses. The patient underwent three additional I&Ds with intraoperative cultures and wound VAC (vacuum-assisted closure) placement due to recurrent abscess formation. *Mycobacterium abscessus* was identified; piperacillin-tazobactam was discontinued, and the patient was started on empiric tigecycline, cefoxitin, and linezolid, awaiting the official susceptibility profile for antimicrobial adjustment. A peripherally inserted central catheter (PICC) line was placed, and the patient was discharged home to continue wound care and antimicrobial treatment.

**Figure 6:**
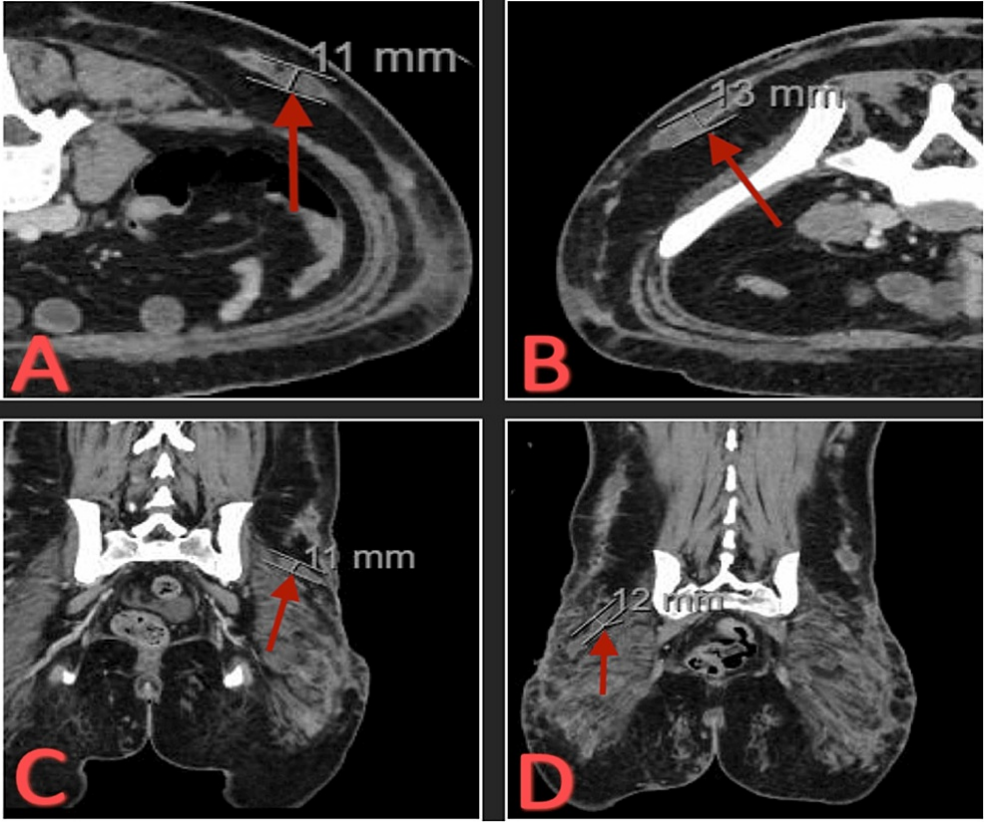
CT abdomen and pelvis with contrast. (A) Transverse view of 11 mm subcutaneous soft tissue micro-abscess. (B) Transverse view of 13 mm subcutaneous soft tissue micro-abscess. (C) Coronal view of 11 mm subcutaneous soft tissue micro-abscess. (D) Coronal view of 12 mm subcutaneous soft tissue micro-abscess.

The patient returned to the hospital due to a worsening infection of the left gluteal region. The patient was admitted for continuation of antibiotic treatment. Preliminary susceptibilities showed resistance to linezolid and clarithromycin with a minimal inhibitory concentration (MIC) >16 mcg/mL. Therefore, linezolid was discontinued, and the patient was started on amikacin and continued on cefoxitin and tigecycline. ART therapy was continued. The patient underwent a fifth I&D due to multiple deep abscesses. Repeat CT of the abdomen and pelvis with contrast showed extensive bilateral, 11-13 mm micro-abscesses throughout subcutaneous soft tissues of the gluteal and lower abdominal wall (Figure 6).

**Figure 7:**
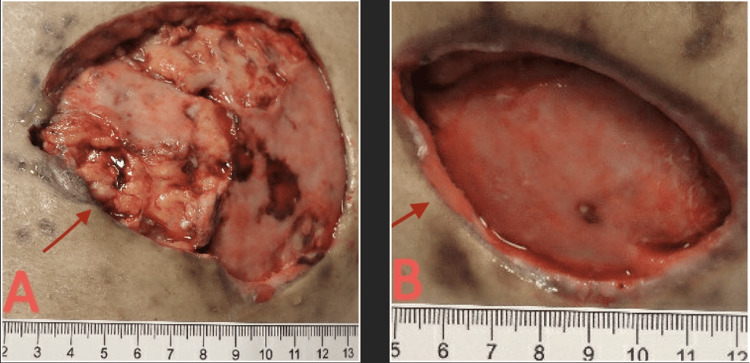
(A) Right wound status post sixth I&D. (B) Left wound status post sixth I&D. I&D, incision and drainage.

The patient underwent the sixth and final I&D (Figure 7).

The patient remained in the hospital for another two months while the comprehensive drug susceptibility testing returned. When the results returned, they showed susceptibility to the current regimen. The patient continued the treatment for a total of approximately four months with adequate clinical response (Table 3).

**Table 3:** Antibiotic susceptibility profile. S: susceptible; I: intermediate; R: resistant; NI: no CLSI interpretive guideline; CLSI: Clinical and Laboratory Standards Institute.

Antibiotics	MIC (mcg/mL)	Interpretation
Amikacin	16	S
Amoxicillin Clavulanic Acid	>32/16	NI
Azithromycin	32	NI
Azithromycin (14 days)	>256	NI
Cefepime	>32	NI
Cefotaxime	64	NI
Cefoxitin	≤16	S
Ceftriaxone	>64	NI
Ciprofloxacin	8	R
Clarithromycin	1	S
Clarithromycin (14 days)	>32	R
Clofazimine	≤0.5	NI
Clofazimine/Amikacin	≤0.5/2	NI
Doxycycline	>16	R
Gentamicin	16	NI
Imipenem	8	I
Kanamycin	16	NI
Linezolid	>16	R
Minocycline	>8	NI
Moxifloxacin	>4	R
Tigecycline	1	NI
Tobramycin	16	R
Trimethoprim/Sulfamethoxazole	>4/76	R

Genotyping returned and showed the pathogen had a positive erythromycin inducible resistance *erm*(41) gene mutation. The *M. abscessus* isolate was negative for acquired macrolide resistance, represented by the *rrl* gene mutation, and negative for resistance to aminoglycosides, characterized by the *rrs* gene mutation (Table 4).

**Table 4:** Gene markers from isolate. *erm*: erythromycin resistance methylase.

Mycobacterium abscessus drug resistance markers	
T28 of erm(41) gene	(+) inducible macrolide resistance predicted
rrl gene	(-) acquired macrolide resistance not predicted
rrs gene	(-) suggests susceptibility to aminoglycosides

**Figure 8:**
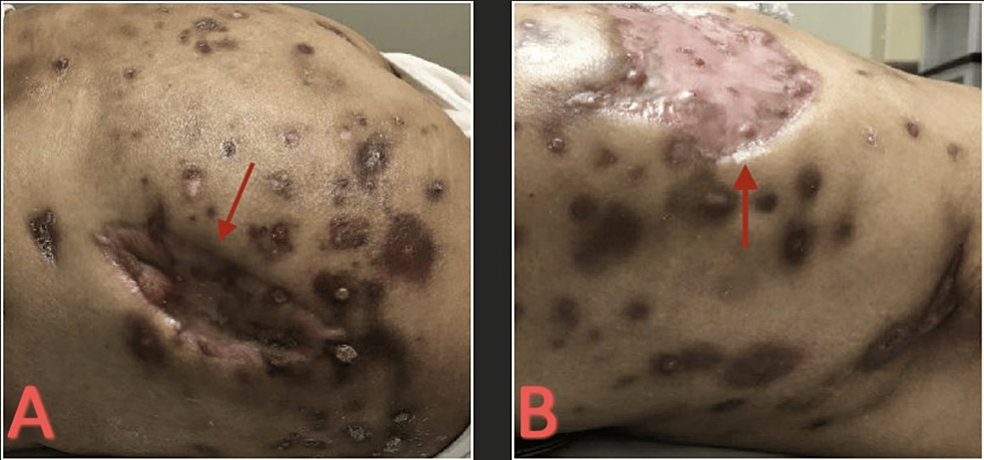
(A) Post-surgical healing of left hip wounds. (B) Post-surgical healing of right hip wounds.

The patient completed treatment and continued to follow up with infectious diseases in the outpatient setting. The patient was prescribed azithromycin 500 mg daily and levofloxacin 750 mg daily for two weeks during an office visit for continued treatment. The patient continued undergoing outpatient wound care with healing left and right hip healing post-surgical wounds (Figure 8).

Case 2

**Figure 9:**
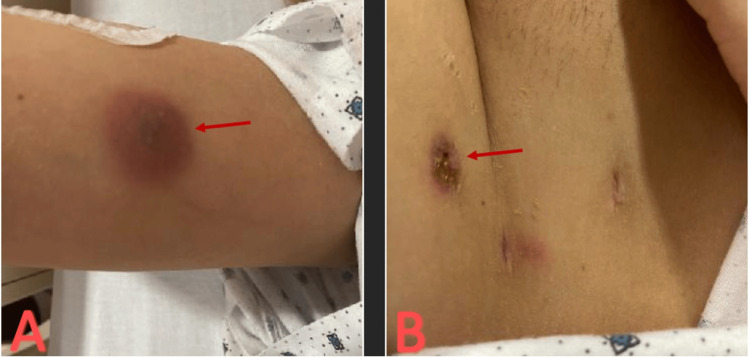
(A) Initial arm lesion. (B) Initial inguinal lesion.

A 28-year-old woman underwent a BBL in Mexico. The patient went to a follow-up appointment with the plastic surgeon without complications. She started to develop fevers, chills, and pain in her gluteal area one month after the procedure. She presented to an outside facility with tender, dark-colored, and indurated lesions in the arm, inguinal area, as well as other areas of the body and was admitted for two months (Figure 9).

Fat was harvested from the abdominal cavity and injected into the gluteal areas with sequelae of disseminated infection after the initial cosmetic surgical procedure. During that hospitalization, the patient was found to have abscesses in bilateral thighs and gluteal areas, right chest wall, and breast. The patient underwent an I&D of the abscesses, where intraoperative cultures were taken. Cultures returned positive for *M. abscessus* and *Pseudomonas aeruginosa*. Susceptibilities of *M. abscessus*, unfortunately, were not obtained. The patient was discharged to a long-term acute facility (LTAC) to complete intravenous (IV) meropenem, amikacin, and clarithromycin.

**Figure 10:**
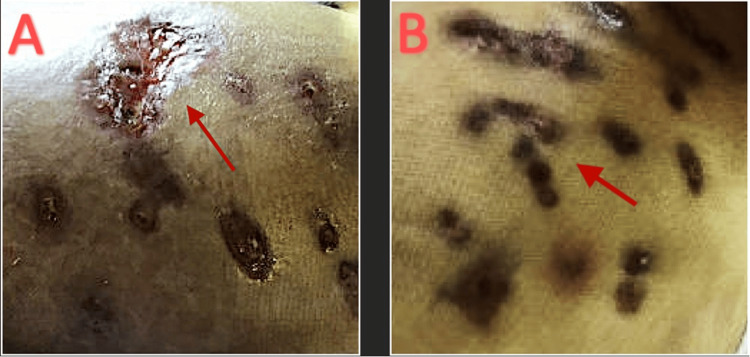
(A) Initial left hip lesions. (B) Initial medial left hip lesions.

Due to the recurrence of the abscesses, the patient presented to our facility for further assessment and care. The patient presented approximately seven months after the initial procedure, previous hospitalization, and IV antibiotic treatment with multiple new abscesses and associated nausea, vomiting, and chills. The patient presented tachycardiac with a heart rate of 120 beats per minute, blood pressure of 146/65 mmHg, and afebrile (37.1°C). On examination, the patient had multiple pink, tender, fluctuant abscesses on her medial and lateral hip (Figure 10).

Addition lesions were observed across the patient’s lower back, gluteal area, and upper thighs. Initial laboratory findings showed anemia and thrombocytosis (Table 5).

**Table 5:** Initial laboratory findings.

Tests	Results	Normal Range
White blood cell count (WBC)	8.12 x 10^3^/UL	4.50-11.03 x 10^3^/UL
Red blood cell count (RBC)	3.85 x 10^6^/UL	3.50-5.50 x 10^6^/UL
Hemoglobin	11.6 g/dL	12.0-15.0 g/dL
Platelets	455 x 10^3^/uL	150-450 x 10^3^/uL
Sodium, serum	141 mmol/L	135-145 mmol/L
Potassium, serum	4.8 mmol/L	3.5-5.1 mmol/L
Chloride, serum	110 mmol/L	98-107 mmol/L
Bicarbonate	22 mmol/L	22-30 mmol/L
Glucose	102 mg/dL	74-106 mg/dL
Creatinine	0.5 mg/dL	0.52-1.04 mg/dL
Blood urea nitrogen, serum	10 mg/dL	7-17 mg/dL
Magnesium, serum	1.6 mg/dL	1.6-2.3 mg/dL
Phosphorus, serum	4.7 mg/dL	2.5-4.5 mg/dL
HIV 1/2 ELISA	Negative	Negative

**Figure 11:**
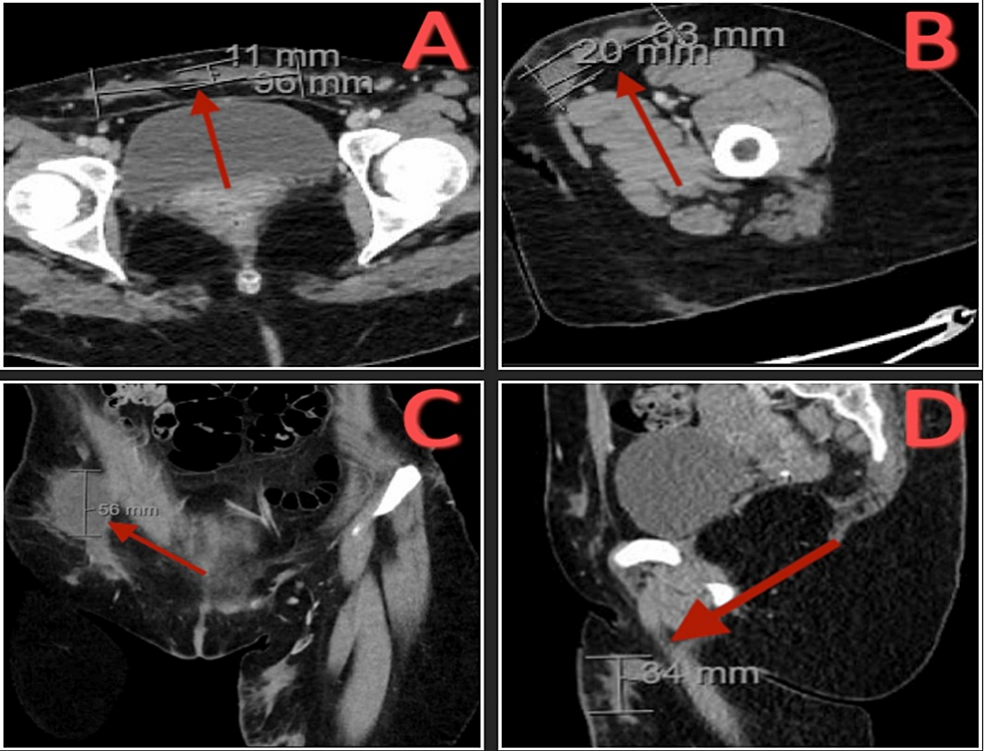
CT pelvis with contrast. (A) Transverse view of an 11 x 96 mm abscess in subcutaneous fat of the lower anterior abdominal wall. (B) Transverse view of a 20 x 63 mm abscess in the subcutaneous fat of the left proximal thigh. (C) Coronal view of 56 mm abscess in the subcutaneous fat of the right hip. (D) Sagittal view of a 34 mm abscess in the subcutaneous fat of the gluteal region.

CT of the pelvis with contrast showed multiple abscesses in the subcutaneous fat of the lower anterior abdominal wall 11 x 96 mm, of the left proximal thigh 20 x 63 mm, of the right hip 56 mm, and of the gluteal region 34 mm (Figure 11).

The patient was empirically started on vancomycin, levofloxacin, and metronidazole, which was changed to amikacin, tigecycline, and cefoxitin.

**Figure 12:**
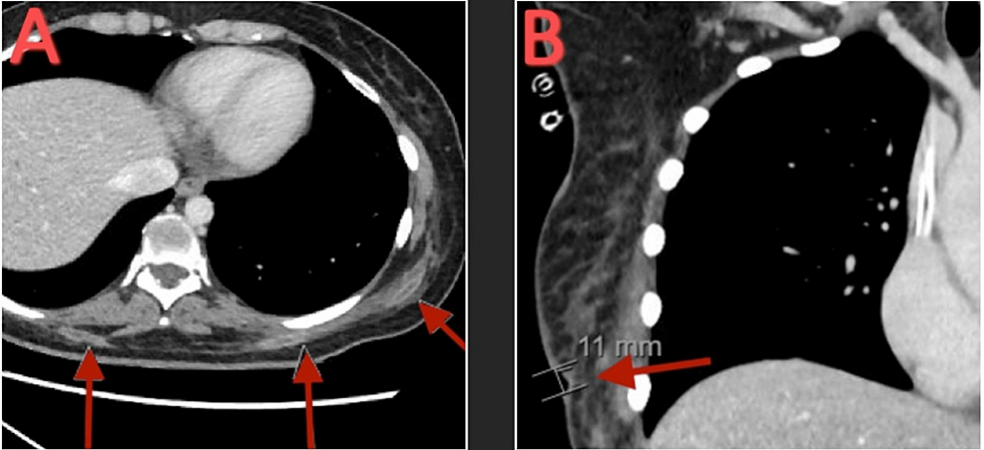
CT thorax with contrast. (A) Transverse view showing multiple tubular, interconnecting, loculated soft tissue abscesses. (B) Coronal view showing a 1.1 cm soft tissue ulcer in the inferior and lateral aspect of the right breast.

Ultrasound-guided catheter drainage led to the drainage of only one abscess in the right lateral gluteus. The patient consented to undergo I&D due to numerous remaining abscesses. The patient underwent a surgical washout on 21 abscesses found in her thigh, abdomen, gluteal areas, hip, and lower back. Intraoperative cultures were collected and sent for further characterization. All cultures remained negative during hospitalization. Post-surgically, a CT thorax with contrast showed multiple tubular, interconnecting, loculated soft tissue abscesses in the bilateral chest wall and upper back with multiple skin openings and surrounding inflammatory fat stranding. It also demonstrated a 1.1 cm soft tissue ulcer in the inferior and lateral aspects of the right breast (Figure 12).

**Figure 13:**
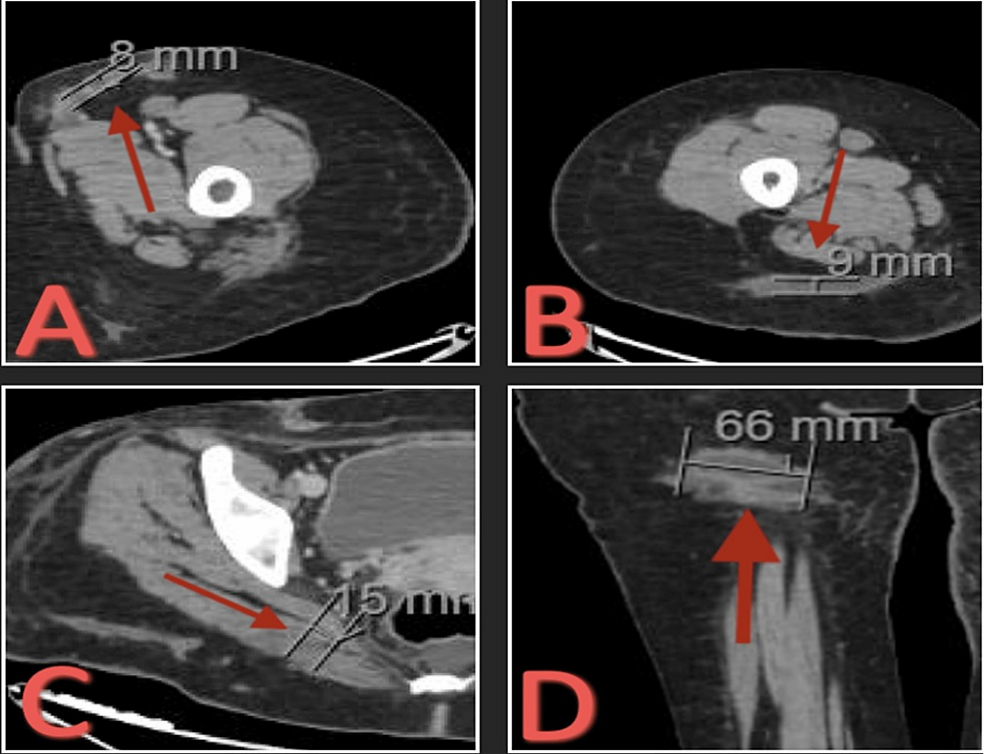
CT bilateral lower extremities with contrast. (A) Transverse view of 8 mm small-sized remnant sub-centimeter collection in the left thigh. (B) Transverse view of 9 mm small-sized remnant sub-centimeter collection in the right thigh. (C) Transverse view of 15 mm small-sized remnant sub-centimeter collection in the right glute. (D) Coronal view of 66 mm small-sized remnant sub-centimeter collection in the right thigh.

A CT of bilateral lower extremities with contrast showed small-sized remnant sub-centimeter collections: 8 mm in the left thigh, 9 mm in the right thigh, 15 mm in the right glute, and 66 mm in the right thigh (Figure 13).

**Figure 14:**
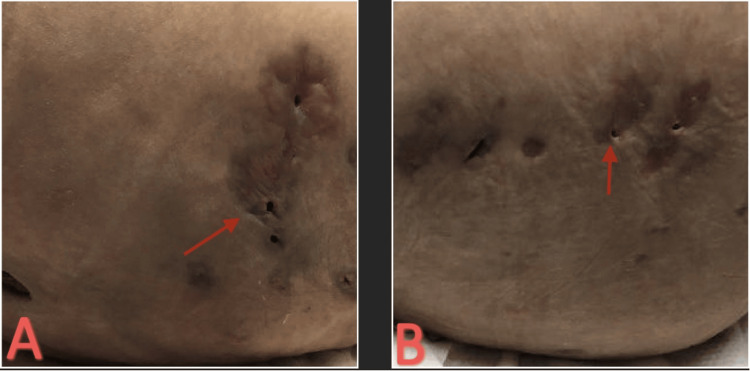
(A) Left hip status post third final I&D. (B) Right hip status post third I&D. I&D: incision and drainage.

The patient underwent two additional I&Ds during hospitalization due to recurrent abscesses for further drainage and source control (Figure 14).

**Figure 15:**
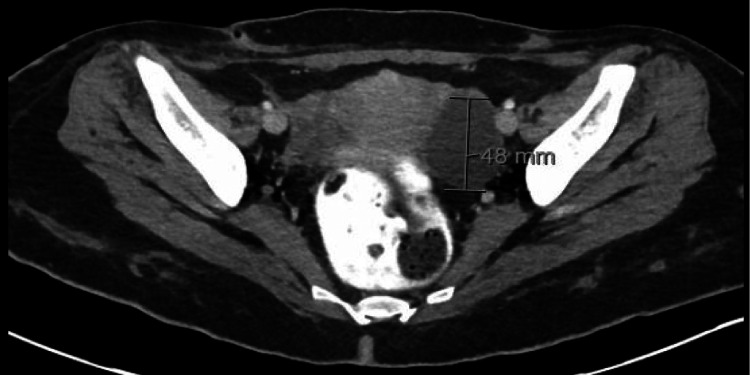
CT abdomen and pelvis with contrast showing a complex 48 mm fluid collection with fistulous tracks within the pelvic cavity.

Before discharge, a repeat CT of the abdomen and pelvis with contrast showed a complex 48 mm fluid collection with fistulous tracks within the pelvic cavity (Figure 15).

No further inpatient I&Ds occurred. The patient was discharged to an LTAC to continue amikacin, tigecycline, cefoxitin, wound care, and close observation. The antibiotic regimen was not modified because there was a strong suspicion of an identical *M. abscessus* isolate as the patient in case 1. Both patients underwent similar cosmetic procedures at the same facility by the same surgeon one week apart.

**Figure 16:**
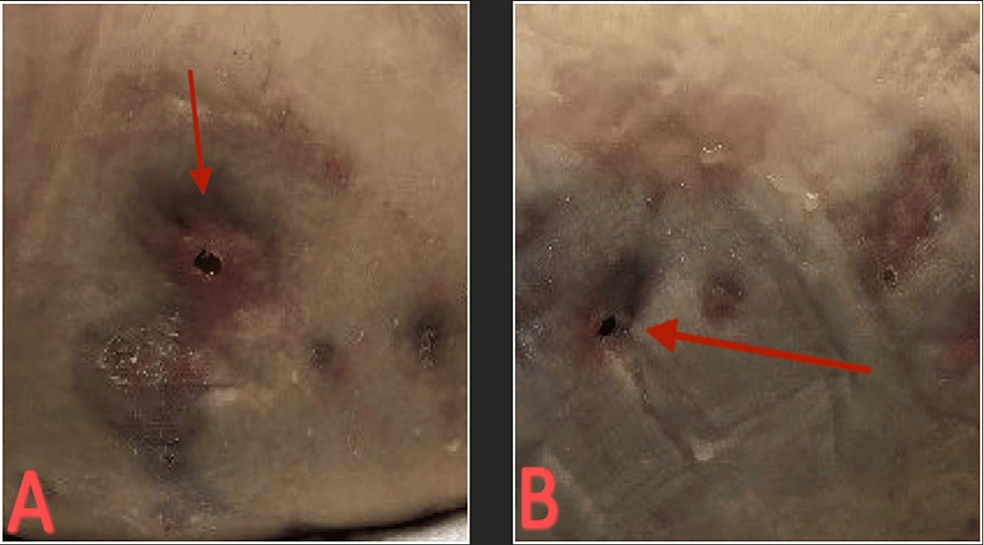
(A) Post-surgical healing of left hip wounds. (B) Post-surgical healing of right hip wounds.

The patient had an outpatient follow-up in the infectious diseases clinic and reported significant improvement in wound healing (Figure 16).

She complained of a decrease in hearing while receiving antimicrobial therapy. Amikacin-associated ototoxicity was diagnosed, prompting its discontinuation and modification of regimen to meropenem, azithromycin, and tigecycline while in the LTAC for a couple of weeks. Antimicrobial therapy was adjusted during clinic follow-up to tigecycline and cefoxitin only for the remaining treatment. The patient completed six months of treatment with the resolution of the soft tissue infectious process.

## Discussion

*Mycobacterium abscessus* complex (MABC) is a group of rapidly growing, multidrug-resistant, NTMs responsible for pulmonary, ocular, and skin and soft tissue infections. It is often regarded as one of the most antibiotic-resistant mycobacteria due to various virulence factors, host interactions, and resistance mechanisms, leaving few therapeutic options [4]. It comprises three subspecies: *M. abscessus subspecies abscessus* (MAB), *M. abscessus subspecies massiliense* (MMA), and *M. abscessus subspecies bolletii* (MBO), which can be further identified by partial gene sequencing: secA1, rpoB, and hsp65, respectively [5]. Subspecies identification is critical for disease management. Subspecies *abscessus* is identified as *M. abscessus* and *M. bolletii*, which have an inducible macrolide resistance gene, erythromycin resistance methylase (*erm*(41)), that results in clinical macrolide resistance. *M. abscessus *is also vulnerable to acquired mutational macrolide resistance via the *rrl *gene. Macrolide resistance has such a profoundly negative impact on *M. abscessus* treatment that preserving macrolide susceptibility with adequate companion drugs such as aminoglycosides (amikacin), cephalosporins (cefoxitin), and/or tetracyclines (tigecycline) is among the highest priorities [6].

*M. abscessus* was first isolated in 1952, with two new related species, *M. massiliense* and *M. bolletii*, discovered in 2006 based on the rpoB gene sequence [7]. It is considered one, if not the most resistant pathogens secondary to its resistance to high levels of chlorine in drinking water, disinfectants such as organic-mercurial substances and alkaline glutaraldehyde, high temperatures, and ability to form biofilms [8]. Among the NTM, MABC is the most common to cause pulmonary disease 3-13%, with *M. abscessus* being the most isolated pathogen [9]. Structural pulmonary abnormalities, such as in patients with cystic fibrosis (CF), have a predisposition for infections with *M. abscessus *[10]. In certain instances, infection with NTM has been misdiagnosed and treated as multidrug-resistant tuberculosis, which delayed appropriate treatment [11]. *M. abscessus* is the most common NTM to cause complicated skin and soft tissue infections (cSSTIs) through direct inoculation via skin barrier breaks and causes a complicated and lengthy treatment course [5].

The pathogenesis of *M. abscessus* is essential to its resistant treatment characteristics. It induces an immune response by activating toll-like receptors and propagating an immune response with excessive inflammatory cytokines such as TNF-𝛼, which causes damage to the host's surrounding tissues [12]. It exhibits both genotypic and phenotypic heterogeneity. Regarding its genotypic heterogeneity, *M. abscessus* and *M. bolletii* express the *erm*(41) gene, leading to its inducible macrolide resistance. This erythromycin ribosome methylase transfers one or two methyl groups to the peptidyl region of 23S rRNA, preventing clarithromycin binding [13]. Other forms of drug resistance are through mutations in the *rrl* gene, *rrs* gene, nucleotide variation at the quinolone resistance determining region (QRDR), synthesis of a class A 𝛽-lactamase, and enzymatic inactivation of tetracyclines through flavin-adenine-dinucleotide (FAD)-inactivating monooxygenase. Mutations in the *rrl* gene, which codes for the 23S rRNA transferase enzyme, can lead to acquired macrolide resistance [9]. Aminoglycoside resistance is acquired through spontaneous single mutations in the *rrs* gene encoding the 16S rRNA [13]. Having nucleotide variation at the QRDR leads to potential resistance to fluoroquinolones [4]. *M. abscessus* also can produce a class A 𝛽-lactamase that creates resistance to many beta-lactams, such as cephalosporins and carbapenems [9]. Cefoxitin is hydrolyzed at a slower rate than other beta-lactams, giving it its therapeutic potential [4]. Enzymatic inactivation of tetracyclines via FAD-inactivating monooxygenase leads to high resistance to doxycycline, but tigecycline resists inactivation, leading to a good therapeutic response [9]. These multiple resistance mechanisms make *M. abscessus *a difficult pathogen to treat.

Regarding phenotypic heterogeneity, it expresses two morphotypes: smooth colonies and rough colonies. During replication, *M. abscesssus* can change between smooth and rough variants, increasing the severity of clinical symptoms [14]. Regarding smooth colonies, this morphotype expresses glycopeptidolipids on its outer cell wall that masks the underlying phosphatidyl-myo-inositol mannosides interfering with pathogen recognition by the host’s innate immune system. The smooth colonies also have the ability for biofilm formation and sliding mobility. A spontaneous mutation in the mmpL4b causes the loss of GLPs on its outer cell wall, causing a morphologic conversion to a rough phenotype, which elicits a vast immune response [15]. These colonies form large serpentine cords, which allow them to evade the immune system and promote the spread to other tissues, abscess formation, and tissue damage [16]. The three subspecies are separated through multilocus sequencing of housekeeping genes, and they possess a single ribosomal operon with conserved genes that include a resistance plasmid. This resistance plasmid is hypothesized to have been acquired through horizontal gene transfer from other pathogens, such as *Pseudomonas* and *Streptomyces* species, contributing to its pathogenesis [9].

Diagnosis of *M. abscessus *infection comprises a combination of clinical, radiological, and microbiologic criteria. Conventional microbiologic diagnosis techniques include staining with Ziehl-Neelsen, culture, and growth. Testing for molecular components of the NTM, such as RNA polymerase (rpoB), gyoB, heat shock protein (hsp65), internal transcribed spacer (ITS), superoxide dismutase (SodA), and 16S 23S rRNA gene spacer amplification, can lead to proper identification and treatment [8]. For appropriate treatment, the Clinical and Laboratory Standards Institute (CLSI) recommends (MICs) susceptibility testing using a multi-antimicrobial panel including amikacin, cefoxitin, clarithromycin, ciprofloxacin, doxycycline, imipenem, linezolid, moxifloxacin, trimethoprim-sulfamethoxazole, and tobramycin [17]. Therapeutic regimens depend on the infection site and typically include macrolide-based combination therapy with parenteral amikacin plus another parenteral agent such as cefoxitin, tigecycline, imipenem, or linezolid. Duration of treatment can be upwards of months to years, followed by a transition to oral antimicrobial therapy [18]. The final selection of antimicrobial therapy is based on susceptibilities. 

Our patients were young women who underwent a BBL procedure in Mexico that resulted in a complicated and extensive hospital course with multiple I&D procedures due to a cSSTI caused by an *M. abscessus* infection. A lack of proper surgical equipment sterilization in combination with poor surgeon sterile technique/training most likely led to the outcome in this case. Literature shows limited reports of skin and soft tissue infections from these types of pathogens undergoing procedures of this kind [19]. It is important to consider NTM after cosmetic surgeries as it has been associated with an increasing cause of numerous cosmetic procedure-related infections worldwide [20]. Hence, the relevance of this case series demonstrates the importance of having this infection differential when assessing a patient who recently had a cosmetic procedure of this type.

## Conclusions

*Mycobacterium abscessus* is a multidrug-resistant pathogen that can cause multiple complications in an infected individual. It is imperative to have a high clinical suspicion of NTM when assessing skin lesions after cosmetic procedures as it has been associated with an increasing cause of numerous cosmetic procedure-related infections worldwide. Inadequate sterilization of surgical equipment, in addition to increased antibiotic resistance, makes preprocedural sterilization necessary to prevent NTM from arising. Additionally, patients may need to be transferred to specialized facilities for further appropriate management of NTM due to a lack of treatment resources in certain facilities if feasible.
